# Optimal Strategies for Controlling Riverine Tsetse Flies Using Targets: A Modelling Study

**DOI:** 10.1371/journal.pntd.0003615

**Published:** 2015-03-24

**Authors:** Glyn A. Vale, John W. Hargrove, Michael J. Lehane, Philippe Solano, Stephen J. Torr

**Affiliations:** 1 Natural Resources Institute, University of Greenwich, Chatham, United Kingdom; 2 South African Centre for Epidemiological Modelling and Analysis, University of Stellenbosch, Stellenbosch, South Africa; 3 Liverpool School of Tropical Medicine, Liverpool, United Kingdom; 4 IRD, UMR 177 Intertryp IRD-Cirad, Montpellier, France; 5 Warwick Medical School, University of Warwick, Coventry, United Kingdom; National Institute of Allergy and Infectious Diseases, UNITED STATES

## Abstract

**Background:**

Tsetse flies occur in much of sub-Saharan Africa where they transmit the trypanosomes that cause the diseases of sleeping sickness in humans and nagana in livestock. One of the most economical and effective methods of tsetse control is the use of insecticide-treated screens, called targets, that simulate hosts. Targets have been ~1m^2^, but recently it was shown that those tsetse that occupy riverine situations, and which are the main vectors of sleeping sickness, respond well to targets only ~0.06m^2^. The cheapness of these tiny targets suggests the need to reconsider what intensity and duration of target deployments comprise the most cost-effective strategy in various riverine habitats.

**Methodology/Principal Findings:**

A deterministic model, written in Excel spreadsheets and managed by Visual Basic for Applications, simulated the births, deaths and movement of tsetse confined to a strip of riverine vegetation composed of segments of habitat in which the tsetse population was either self-sustaining, or not sustainable unless supplemented by immigrants. Results suggested that in many situations the use of tiny targets at high density for just a few months per year would be the most cost-effective strategy for rapidly reducing tsetse densities by the ~90% expected to have a great impact on the incidence of sleeping sickness. Local elimination of tsetse becomes feasible when targets are deployed in isolated situations, or where the only invasion occurs from populations that are not self-sustaining.

**Conclusion/Significance:**

Seasonal use of tiny targets deserves field trials. The ability to recognise habitat that contains tsetse populations which are not self-sustaining could improve the planning of all methods of tsetse control, against any species, in riverine, savannah or forest situations. Criteria to assist such recognition are suggested.

## Author Summary

We employed a deterministic model to simulate the efficacy of various ways of using the tiny, ~0.06m^2^, insecticide-treated targets recently recommended as replacements for the larger, ~1m^2^, types previously used to control riverine species of tsetse fly, the main vectors of sleeping sickness in humans. Results suggested that in many situations the use of tiny targets at treble the normal density for a third of the normal time could be the most cost-effective strategy for rapidly reducing or eliminating tsetse populations, so helping with disease control. In deciding whether to aim for local control or elimination, and in planning the operations, it would be highly advantageous to distinguish those parts of the tsetse infestation that support self-sustaining populations, and those containing populations that cannot be sustained unless supplemented by immigrants. Sorts of information that can help to assess the type of sustainability in field habitats are identified. These findings can assist the planning of any method of tsetse control used against any species of tsetse, including those important as vectors of livestock disease.

## Introduction

Tsetse flies (*Glossina* spp.) occur in 36 countries of Africa where they transmit species of *Trypanosoma* which cause the potentially fatal diseases of sleeping sickness in humans and nagana in domestic stock [1,2]. Vector control can be important in managing these diseases [2]. Of the several techniques recommended for this the cheapest and simplest is the use of pyrethroid-treated cattle [3]. However, this technique is applicable only where the numbers and distribution of cattle are adequate, and where the veterinary authorities approve the treatments. In other circumstances it can be cheapest to use insecticide-treated screens of cloth, called targets [4], as an alternative or supplement to cattle baits. For many years the targets that have been employed against tsetse in either riverine, savannah or forest areas have consisted of screens of ~1m^2^ that are maintained in steady effectiveness for a year or more, through visits every few months to repair or replace damaged or stolen targets and to clear vegetation growth [4].

Recently, however, field experiments [5] and theoretical studies [6] have shown that much smaller targets, of ~0.06m^2^, are especially suitable for use against those particular tsetse that are the main vectors of sleeping sickness, *i*.*e*., riverine species such as *Glossina fuscipes fuscipes*, at least when the flies are occupying their common habitat consisting of strips of woodland bordering rivers or lakes. These tiny targets are convenient to deploy and relatively cheap to manufacture [3], so that they have reinforced the interest in tackling sleeping sickness by vector control [7,8]. They also suggest a need to reconsider several aspects of vector control strategy. First, since the tiny targets are cheap they could be regarded as disposable, so avoiding the costs of retrieving them when the target operation is over. Second, the cheapness could allow the targets to be deployed at increased density, provided control were then so rapid as to be achieved before maintenance visits were required. Such a policy would be best served if the target deployments took place at the start of seasons in which the risk of floods and the degree of vegetation growth were minimal. Third, we need to intensify the debate about whether to aim for local elimination of tsetse, as against the mere reduction of local abundance [3].

Certainly the technical ideal in any locality is to eliminate the flies in a once-off operation, but that requires the tsetse to be isolated naturally in an area small enough to be covered all at once [9,10]. Usually, however, operations must be conducted in large infestations so that only part can be tackled at any one time. In that case, as soon as the control operation is over the treated area can be re-populated by flies invading from adjacent untreated areas. One means of dealing with this problem is to establish artificial barriers to invasion, as exemplified by deploying baits in the invasion route [11,12,13], perhaps as temporary expedients before renewed control operations move into the invasion source [14]. Such barriers are likely to be most effective when tackling tsetse in riverine situations where the linear habitat reduces tsetse mobility [6,15] and ensures that invasion can occur only along the river, not from all directions as commonly happens in large blocks of savannah or forest.

However, the cheaper, simpler and quicker our control methods become the less important it is to eradicate tsetse from whole infestations or to maintain perfect and permanent barriers to invasion. Instead, the more appropriate strategy could be to hit the flies in quick local operations that are not expected to eliminate tsetse completely, but which can be repeated readily when the tsetse population recovers to unacceptable densities. The credibility of this strategy is supported by models showing that trypanosomiasis in livestock can be tackled effectively by tsetse control that stops well short of elimination [16], and that the required levels of control to deal with human trypanosomiasis can be even more modest [17,18]. These indications are important because tsetse control is primarily a means of disease prevention, not an end in itself.

The readiness with which a suitable level of tsetse control can be achieved will depend on the way that the habitats in or near the operational area affect the natural population dynamics of tsetse. For example, the feasibility of countering invasion will improve if the invasion source is poor habitat that can support only low densities of tsetse, thus reducing the invasion pressure. The invasion problem becomes even less important if the tsetse population in habitats adjacent to the operational area are sustainable only because they are supplemented by flies immigrating from the operational area [19]. The ability to benefit from these matters is likely to be greatest when tsetse are limited to narrow riverine situations. This is because the more restricted the invasion route the greater the chances of locating the operational area where the immediate source of invading flies is relatively poor habitat offering a narrow invasion front [13].

The upshot of all of the above matters is that the planning of the most cost-effective control of tsetse in riverine situations should consider a wide range of options for the intensity, duration, scale and spatial relationships of operations, especially now that the efficacy of tiny targets is recognised. To explore these options we first simulated different sorts of habitat associated with distinctive population dynamics of tsetse. We then modelled the efficacies of various strategies for using targets in a variety of riverine scenarios involving such habitats and different types of invasion problem.

## Methods

### Ethics

There were no ethical issues since all work was theoretical.

### Model

The tsetse population was considered to be confined entirely to a narrow strip of riverine habitat although, in accord with field conditions [20], it was allowed that various segments of the strip varied in the suitability of the habitat they provided. The daily abundances of various components of the tsetse population in each 1km-long segment of the strip were tracked deterministically in Microsoft Excel spreadsheets, in essentially the same way as in the Tsetse Muse model, detailed by [21] and available at www.tsetse.org. However, the present model differed from Tsetse Muse in that the death rates that targets imposed on adult tsetse were not always constant but were allowed to vary from day to day, to reflect changes in the efficacy and/or abundance of targets during the control campaign.

#### Standard population of tsetse

This population occupied uniformly good habitat and consisted of 2500 adult males/km^2^ and 5000 adult females/km^2^. Daily death rates of adults were age-dependent, averaging 0.0614 for males and 0.0307 for females, with maximum adult life spans of 89 and 178 days, respectively, consistent with field evidence that females live longer than males [22]. The death rate of eggs and larvae (1st-3rd instar) per larval period was 0.05. Males were sexually mature at five days and females at three. The first larva was produced at age 16 days and the interlarval period was nine days. The pupal period was 28 days for males and 26 days for females, with a death rate of 0.25 per period. Males and females emerged in equal numbers.

#### Density dependence

Natural death rates of adults, pupae and eggs/larvae in each segment of the strip declined linearly with reductions in population density there, to be steady at 75% of standard rates when the population density was ≤10% of standard. The density reference for the rates of adults and eggs/larvae was the abundance of all adults, and for pupae it was pupal abundance. Females found mates with a daily probability of 0.10 if there was only one mature male/km^2^. Provided the abundance of such males was not limiting, an isolated population could increase up to 8.4 times per year, as observed for isolated field populations [22].

#### Poor habitats

Some places, in which no tsetse could survive and none ventured, were called "no-go areas". Other, less extreme, sorts of poor habitat were areas that contained tsetse but where the density-specific and age-specific death rates of at least one sex or life stage of the tsetse population was greater than standard in each 1km-long segment of the poor habitat. Two main types of poor habitat were simulated. In the first type, called poor habitat S (self-sustaining), all of the death rates applicable at standard density were increased by 1.3 times. This lowered the abundance of tsetse, but density dependence was then sufficient to ensure that the death rates declined enough to allow the population to maintain a new stability, whereas if the death rates at standard density had been increased just a little more, by 1.4 times, the population would have declined slowly to extinction. In the second type of poor habitat, called type NS (non-self-sustaining), all death rates were increased 2.0 times, so ensuring that the population was far from sustainable unless it were regularly supplemented by natural immigration of tsetse from better habitat nearby. Populations that are not self-sustaining are known to occur, as evidenced by the fact that they disappear when flies in the immigration source are eliminated [19,23]. The stabilized distribution of tsetse between various habitats prior to any control was produced by seeding each 1km-long section of habitat with an estimate of the stable population there, and then performing 10,000 iterations of daily movements, births and deaths.

In keeping with field experience [24,25], it was allowed that in the dry season some poor habitats along river courses could become no-go areas, represented for example by headwaters and small tributaries where the vegetation is sparse and the stream bed dries out seasonally. However, in all other habitats it was taken that tsetse showed no seasonal variation in abundance. This is consistent with observation that the apparent densities of riverine tsetse associated with permanent water bodies do not exhibit the gross seasonal changes usually occurring with savannah tsetse. It is also consistent with the fact that many riverine tsetse experience no particularly dry season, or only short dry seasons between two rainy periods each year.

#### Mobility and "reflective" contacts

Tsetse movement was between adjacent segments along an East-West strip of habitat. Unless stated otherwise, the daily rate of transfer of tsetse between adjacent segments of the same type of habitat was set so that the root mean daily displacement [6], measured over 30 days, averaged 125m for males and 269m for females, to suit the field data showing that female tsetse are more mobile than males [11,26]. However, it was sometimes allowed that when flies in a segment of good habitat reached its contact with a segment of poor habitat they were "reflected" back to the good habitat instead of entering the poor. This phenomenon was simulated by reducing the probability of tsetse transfer to the poor habit, while leaving untouched the transfer in the reverse direction.

#### Control methods

All simulated control was performed by baits that were allowed to show one or both of two stages of efficacy before the targets became completely ineffective. First, there was a level stage in which the daily death rates imposed on adults were assumed constant from day to day because all of the targets had recently been deployed, so that each was still in place and was at maximum efficacy. Second there was a declining stage in which the imposed death rates dropped linearly from day to day, to become zero when the stage was over. The decline was envisaged as caused by chemical deterioration of the insecticide or physical loss or damage of the target itself due to say floods, fire, animals or theft.

Various sorts of control strategy were compared with what was called normal steady control. This involved a continuous level stage in which targets were deployed at densities sufficient to kill 5% of the adult male and female tsetse populations per day, which is usually associated with >99.99% control of an isolated population in a year [4]. The 5% kill rate is produced in the field by tiny targets placed at ~20/km of river, or ~5/km^2^ of total land surface. The normal steady control was compared with what was called standard seasonal control in which targets were deployed at about three times the normal density, sufficient to raise the daily death rates to 15% in a level stage lasting three months, before a declining stage lasting another three months, after which the targets were completely ineffective for the final six-months of the year. For all control measures, the targets were considered to be deployed in a single day, and all death rates mentioned refer to those in the level period.

#### Critical level of control

No attempt was made to simulate the way that reduced tsetse densities impacted on the incidence of sleeping sickness since this is subject to many variables [16,17]. However, it can be taken that any given level of tsetse control will produce simultaneously a comparable reduction in the biting rate. The immediate impact on the incidence of disease will depend a little on the associated change in the age structure of the tsetse population, since this will affect the proportion of flies old enough to be infective [27]. Nevertheless, it can be taken that the incidence of disease would drop immediately by roughly the degree of tsetse control. In the longer term the reduced incidence will lead to reduced prevalence, and hence to a downward spiral in the risk of new infections of tsetse and hosts. This expectation that the control of sleeping sickness will be more marked than the control of tsetse is exemplified by the indication that a 75% decrease in tsetse density can lead to a 95% reduction in disease risk in a year, and the virtual absence of risk after two years [17]. Hence, in interpreting present results it was considered that a 90% reduction of tsetse density, maintained for about a year, was the critical degree of control needed for a worthwhile impact on the risk of sleeping sickness.

## Results

### Types of poor habitat

To explore various types of poor habitat, each was considered never to degenerate into a seasonal no-go area and was modelled as a strip at least 40km long adjoining end to end with a similarly long strip of good habitat.

#### Increased death rates

**Fig 1:**
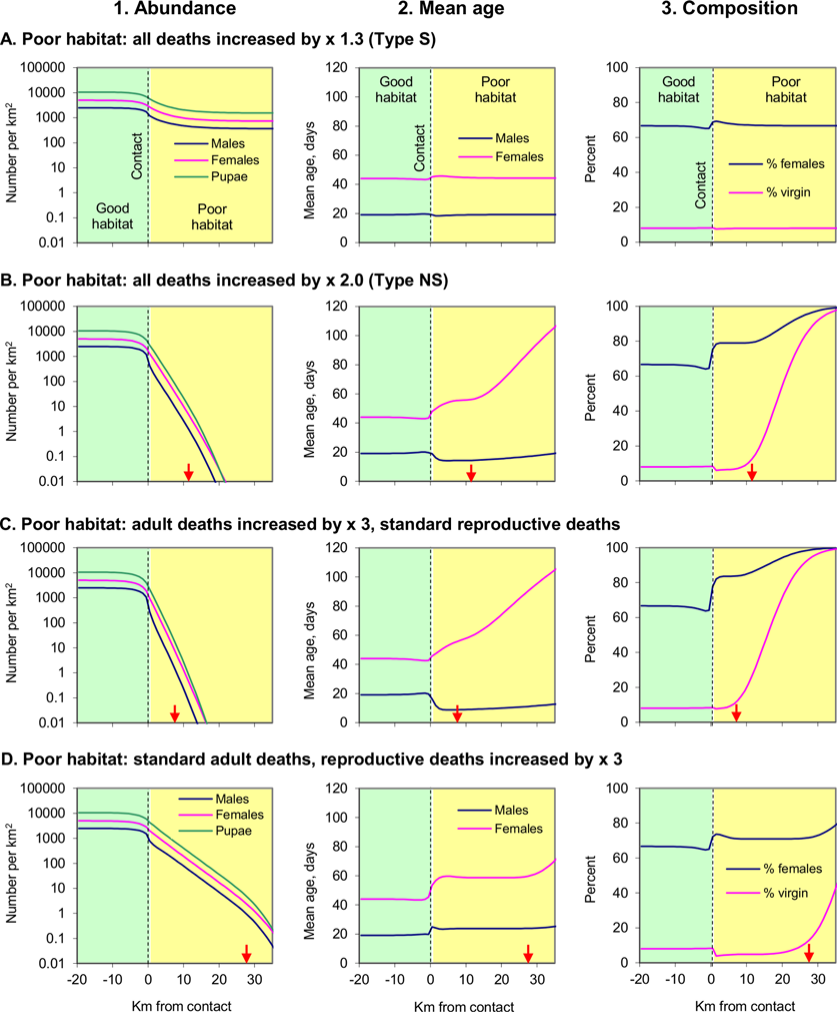
Population data associated with adjoining habitats. Abundances (1) and mean ages of adult male and female tsetse (2), and percents (3) of females among all adults and of virgins among all females, at various distances from the contact between good and poor habitats, when the poor was associated with: all deaths increased 1.3 times (A), all deaths increased 2.0 times (B), adult deaths increased 3.0 times but standard reproductive deaths (C), and standard adult deaths but reproductive deaths increased 3.0 times (D). Good and poor habitats are shaded green and yellow, respectively. Red arrows show where mature males became so scarce that the daily probability of a mature virgin female finding a mate fell below 10%. Absence of a red arrow signifies no such fall in mating-finding success.

It was taken first that the poor habitats contained relatively few tsetse because the death rates of all or some of the population components were enhanced, and not because tsetse showed any aversion to entering the poor habitat. Two phenomena emerged in these simulations, irrespective of what death rate was increased, and by how much (Fig. 1). First, the population in the good habitat at a few kilometres from the contact was greater than in the good habitat closer to the contact. This was because many of the flies in good habitat beside the contact diffused out to the poor habitat and their numbers could not be replenished by opposing transfers from the relatively sparse population in the poor habitat. Second, although the population of tsetse in the poor habitat within a few kilometres of the contact was reduced, it was not exceedingly low there. This was because the population in the poor habitat was supplemented by flies moving in from the good habitat.

The main differences between the numbers of tsetse associated with the various sorts of poor habitat occurred at about 5km or more from the contact. Thus, when the poor habitat was of type S, *i*.*e*., not so bad as to be unable to support a self-sustaining population, the abundance of tsetse soon levelled at various distances far from the contact (Fig. 1, A1). In contrast, when the poor habitat was so bad that the populations in them were not self-sustaining, the abundance of tsetse fell off steadily with increasing distance from the contact (Fig. 1, B1, C1, D1). However, the rate at which this occurred was greater when the poor habitat involved increased deaths of adults (Fig. 1, B1, C1), as against involving only the increased deaths of eggs/larvae and pupae, *i*.*e*., reproductive deaths that reduced the emergence of new adults (Fig. 1, D1).

Other differences between the types of poor habitat involved the way that the age structure of their populations changed on moving away from the contact. With the poor habitat of type S, the mean age of adult female tsetse (Fig. 1, A2) was much more level than in other poor habitats where the populations were not self-sustaining (Fig. 1, B2, C2, D2). This was because the non-self-sustaining populations contained a high proportion of females that had moved into the area from the relatively dense populations nearer the contact—such movement took time so that the females that completed it were relatively old. The mean age of males was affected somewhat differently in that with the non-self-sustaining populations the mean age declined on moving further from the contact if the lack of self-sustainability were due largely or entirely to increased death rates of adults (Fig. 1, B2, C2). This was because the daily mobility was less for males than for females, and because the males were shorter lived, so having fewer days to move into the poor habitat and compensate for the many males dying there. When the poorness of the habitat was due entirely to increased reproductive deaths, not adult deaths, the movement of adult males was effective in increasing the mean age, albeit still not so much as with females (Fig. 1, D2). Increased reproductive deaths might occur, for example, if there were few suitable sites for pupae.

Further differences between the tsetse population in the various types of poor habitat concerned the percent of females among all adults. This percent was hardly changed in the self-sustaining population (Fig. 1. A3). However, it increased substantially with the non-self-sustaining populations (Fig. 1, B3, C3, D3), again due to the relatively great mobility of females. Hardly surprisingly, the percent of virgin females was very high where the population density of males was very low, *i*.*e*., in non-self-sustaining populations far from the contact (Fig. 1, B3, C3, D3).

#### Reflective contacts

**Fig 2:**
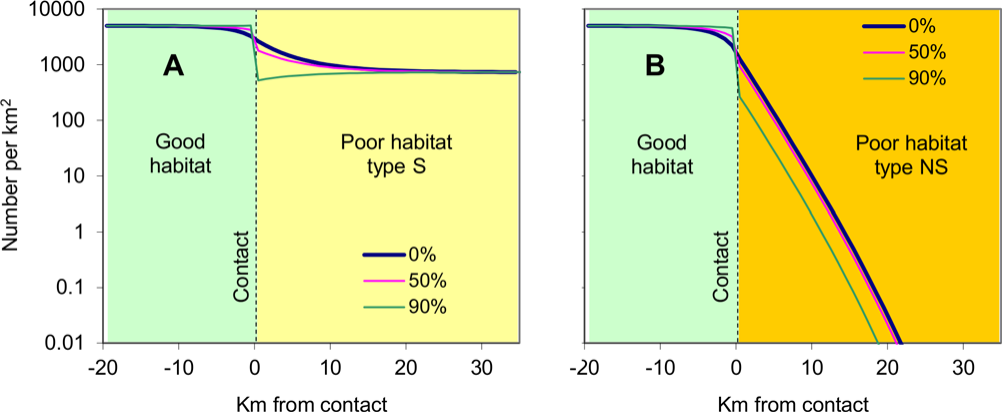
Population distribution associated with adjoining habitats involving reflective contacts. Abundances of adult female tsetse at various distances from the contact between good and poor habitats when the poor habitat was type S (A), or type NS (B), with reflectances of 0%, 50% and 90%. Reflectance applied only when the flies were heading towards the poor habitat.

Making the contact reflective had generally modest effects, as illustrated by applying a 50% or 90% reflectance with contacts involving good habitat adjacent to poor habitats S or NS (Fig. 2). First, the greater the reflectance the greater the abundance of flies in the good habitat near the contact, since fewer tsetse vacated that position. Second, an increased reflectance decreased the abundance of flies in the poor habitat near the contact because fewer flies entered that place. Further from the contact the tsetse population in poor habitat S was unaffected (Fig. 2 A) and even with the poor habitat of type NS the effect was not to alter the rate at which tsetse abundance declined with distance, but only to change by 1–3km the point at which the population reached very low densities.

The reflectance of the contact had no material effect on the distinctive composition of the tsetse populations that were not self-sustaining. For example, when poor habitat NS adjoined good habitat and the reflectance of the contact was 90%, the percent of females in the adult population and the mean age of females in the poor habitat at 3.5km from the contact were both high at 79% and 53 days, respectively. These figures compare with the lower ones of 66% and 44 days for the population in good habitat at 3.5km from the contact.

### Control of isolated populations

Isolated populations were considered to be those occurring in places entirely surrounded by a no-go area, or those in the centre of very extensive operations, covering say ≥50km of river so that the centre is ≥25km from any invasion front. In isolated situations it is possible to achieve the ideal of local elimination of tsetse. Hence, in studying such situations most attention was directed at identifying what was needed for the ideal.

#### Normal steady control

**Fig 3:**
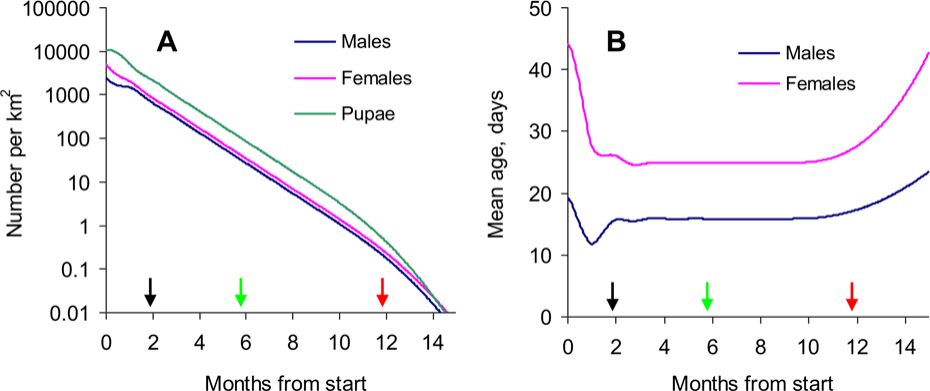
Time course of population change during standard steady control in good isolated habitat. Effects of targets that kill 5% of the adult population per day, and are maintained continuously at maximum performance against a standard population of tsetse in an isolated area of good habitat: (A) effects on the numbers of pupae and adult males and females per km^2^, and (B) mean age of adult males and females. Arrows indicate when the control of adult male plus female tsetse reached 90% (black arrow) and 99% (green), and when the population began to collapse due to inadequate availability of mates (red).

When normal steady control was applied against the standard tsetse population in good habitat, there was a varying rate of decline in adult numbers during the first month or so (Fig. 3, A). This was because the targets did not immediately affect the numbers of adults emerging from pupae, and because time was required to establish a new stability in the age structure of the adult population (Fig. 3, B). Since females were normally longer lived than males, their numbers were most affected by the daily toll at targets, so that the percent of females in the adult population dropped from the initial 67% to only 56% after 40 days. During the next nine months the rate of population decline and the age and sex structures remained virtually constant. Subsequently, however, the population started to drop more rapidly (Fig. 3, A) and the mean age of the adults increased (Fig. 3, B). These effects were due to the abundance of mature adult males approaching the critical level of 0.16/km^2^ needed to ensure that females found a mate in good time. Failure to find mates impeded reproduction, so that fewer young adults were produced. At this point, when the number of adult males and females remaining was about 0.01% of the initial population, the reduction in tsetse abundance due to the targets was supplemented by the natural collapse of the population.

A substantial impact on the threat of sleeping sickness could be expected after 87 days, when the combined density of adult males and females had dropped by 90% (Fig. 3, A). Moreover, at that time the mean age of tsetse had dropped by four days for males and 20 days for females (Fig. 3, B), representing a decrease in mean age of 19% and 44%, respectively. This indicates that a reduced proportion of the tsetse population would be infective, thus decreasing further the transmission of disease. By 173 days the threat of the disease could be taken as almost gone since the total population of adult tsetse had declined by 99% and the mean age of tsetse was still low.

In all subsequent modelling it was found, in accord with above results (Fig. 3), that control measures impacted somewhat differently on adult males and females in the early weeks of control, but that the overall degree of population decline was much the same for each sex. The data for each sex were therefore pooled for reporting. It was taken that an isolated population was effectively eliminated when the population of adults dropped below 0.01% of the standard population density. At such low population densities, involving <1 female per km^2^, the probability of extinction rapidly approaches 100% as adult female mortalities approach 3% per day—even in the unlikely event that the surviving females are inseminated [28].

#### Increased target density and reduced upkeep

**Fig 4:**
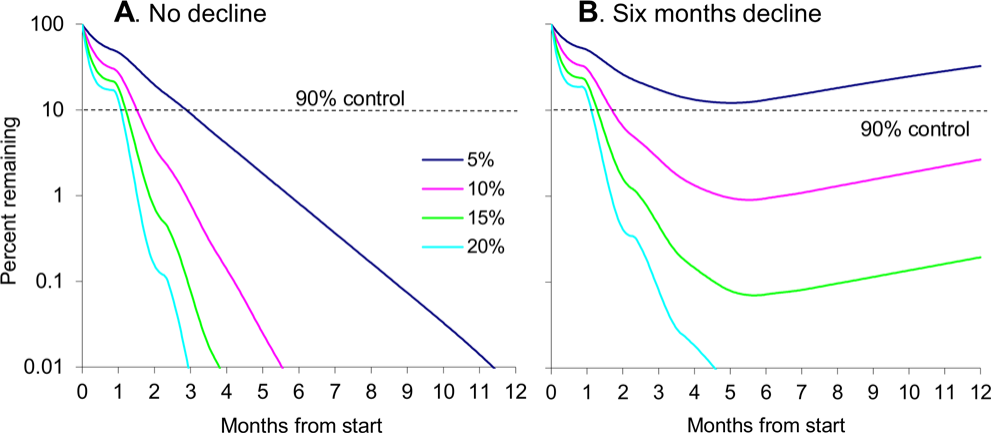
Time course of population change during various sorts of control in good isolated habitat. Percent of initial tsetse population remaining at various times after the deployment of targets at densities sufficient to get initial kill rates of 5–20% of tsetse per day in good isolated habitat, under conditions in which: (A) the efficacy of the targets did not decline, or (B) declined steadily to zero in six months. The population is regarded as the total of adult males and females, and can be taken as locally eliminated when the percent remaining dropped below 0.01%.

When the density of targets deployed against the standard population in good habitat was increased, to raise the imposed daily death rates by up to four-fold, the speed of control increased markedly provided the targets were kept in good order throughout (Fig. 4, A). The time needed to produce a 90% control with steady death rates of 10%, 15% and 20% was 46, 37 and 33 days, respectively, as against the 87 days with the standard steady death rate of 5%. As a rough rule of thumb, once the new age structure of the tsetse population was stabilised after about a month, the daily rate of decline in adult abundance was proportional to the imposed death rate.

However, the speed of population reduction was much decreased (Fig. 4, B) if it were taken that the targets were not well maintained and began to deteriorate immediately, so that their kill rate declined linearly from day to day, starting on the first day after target deployment and reaching zero after six months. In such circumstances it was only with an initial death rate of 20% per day that population reduction was still fast enough to cause local elimination. With the lower initial rates the population started to recover within a week of the rate dropping to 1% per day. Nevertheless, although the initial death rates of 10% and 15% did not lead to local elimination, they were sufficient to ensure that the population was still controlled by 97.3% and 99.8%, respectively, at the end of the year. It was only with the 5% death rates that control never reached the critical level of 90%.

The results of other simulations of control in good habitat (Table 1) showed that local elimination could be achieved with initial daily death rates lowered to 15% or 10%, provided the rate did not decline to zero until nine or twelve months, respectively. However, even with initial kill rates of 20% per day, local elimination could not be achieved by decline stages as short as three months unless that period were preceded by a stage of level efficacy that lasted for at least two months. More satisfactory results were obtained by repeating the control measures in the same seasons of successive years (Table 2). For example, local elimination was achieved in two years by an initial kill rate of 10% if the three month decline period in each year were preceded by level period of at least two months. The number of years needed to achieve the elimination increased markedly as the duration of the level period or the initial density of targets decreased, it being impossible to eliminate the flies in any number of years if the initial kill rate were only 5% and there were no level stage before the three-month decline stage.

**Table 1:** Control achieved by different kill rates imposed in various seasonal patterns, in good isolated habitat.

Months per stage		Initial daily kill rate			
Level	Declining	5%	10%	15%	20%
**0**	**12**	2.01	0.00 (9)	0.00 (5)	0.00 (4)
**0**	**9**	8.76	0.14	0.00 (6)	0.00 (4)
**0**	**6**	32.52	2.66	0.19	0.00 (5)
**0**	**3**	73.32	38.30	13.93	4.37
**1**	**3**	46.68	7.51	0.93	0.10
**2**	**3**	23.27	1.15	0.03	0.00 (4)
**3**	**3**	9.36	0.17	0.00 (4)	0.00 (3)
**4**	**3**	3.52	0.00 (7)	0.00 (4)	0.00 (3)

Percent of the initial population density of tsetse remaining a year after deploying targets sufficient to impose an initial daily kill rate of 5–20%, followed by various months duration of the level stage and declining stage of target efficacy. Where the percent remaining is shown as 0.00, the figures in parentheses are the rounded-up number of months required to bring the percent below 0.01, *i*.*e*, the population's collapse point.

**Table 2:** Elimination times associated with different kill rates imposed in various seasonal patterns for a number of years, in good isolated habitat.

Duration of level stage	Initial daily kill rate			
	5%	10%	15%	20%
**0 months**	-	10	4	3
**1 month**	21	4	2	2
**2 months**	6	2	2	1
**3 months**	4	2	1	1

Number of years required for local elimination of an isolated tsetse population using kill rates of 5–20% at the start of each year, with different durations of the level stage of target efficacy, and with the declining stage set at three months. In the level stage the efficacy of each target is at its maximum each day. In the declining stage the daily efficacy of the targets declines linearly to zero. Where "-" is shown the population could not be eliminated in any number of years.

#### Poor habitat

All of the above simulations referred to control in good isolated habitat. In considering control in poor isolated habitat it was necessary to deal only with those types, such as type S, that supported a self-sustaining population, since isolated habitat that does not do so cannot contain tsetse.

The main lesson was that poor habitat containing much reduced densities of tsetse were usually not associated with correspondingly great reductions in the time needed to eliminate the population. For example, with poor habitat S, which contained a stable self-sustaining population that was only one-seventh as dense as the standard density, the elimination time with standard steady control was 227 days, as against 342 days with the standard density in good habitat. For the standard seasonal control the corresponding figures were 87 days and 119 days respectively. In all cases the time to elimination was reduced by less than half.

The benefit of working in poor habitat was particularly marked only in those cases in which a seasonally applied control measure was not quite effective enough to eliminate tsetse from good habitat. For instance, in good habitat the tsetse could not be eliminated if the initial kill rate was 15% per day and then declined steadily over the next six months (Fig. 3, A). However, when that control measure was applied in poor habitat S the population was eliminated after 121 days.

### Control of non-isolated populations

Whereas the above simulations dealt with operational areas that embraced the whole tsetse population, the next simulations considered operations covering only part of it. In such circumstances it was pertinent to distinguish two sorts of area: (i) the intervention area in which targets were always deployed for at least some of the time and where it was intended to reduce tsetse densities substantially, and (ii) the invasion source which was separated from the intervention area by the invasion front and which might or might not have targets in it to counter the invasion pressure.

Additionally, it was pertinent to recognise two main sorts of invasion pressure. First, there is sustained pressure from a source containing a self-sustaining population of tsetse. In this case the continual invasion means that tsetse cannot be eliminated, so that the success of operations must be measured against the 90% level of critical control. Second, there is non-sustained pressure from a source where the population can be maintained only if supplemented by immigration from the intervention area. In this second case the population in the invasion source would decrease when the flies in the intervention area declined, so that treatment of the intervention area alone could eliminate tsetse from that area and the invasion source [19, 23]. Thus, with non-sustained invasion it is feasible to adopt local elimination as the criterion for success. With these yardsticks in mind, we explored the relative efficacies of normal steady control and standard seasonal control in operational areas subject to the two types of invasion pressure.

### Sustained invasion pressure

**Fig 5:**
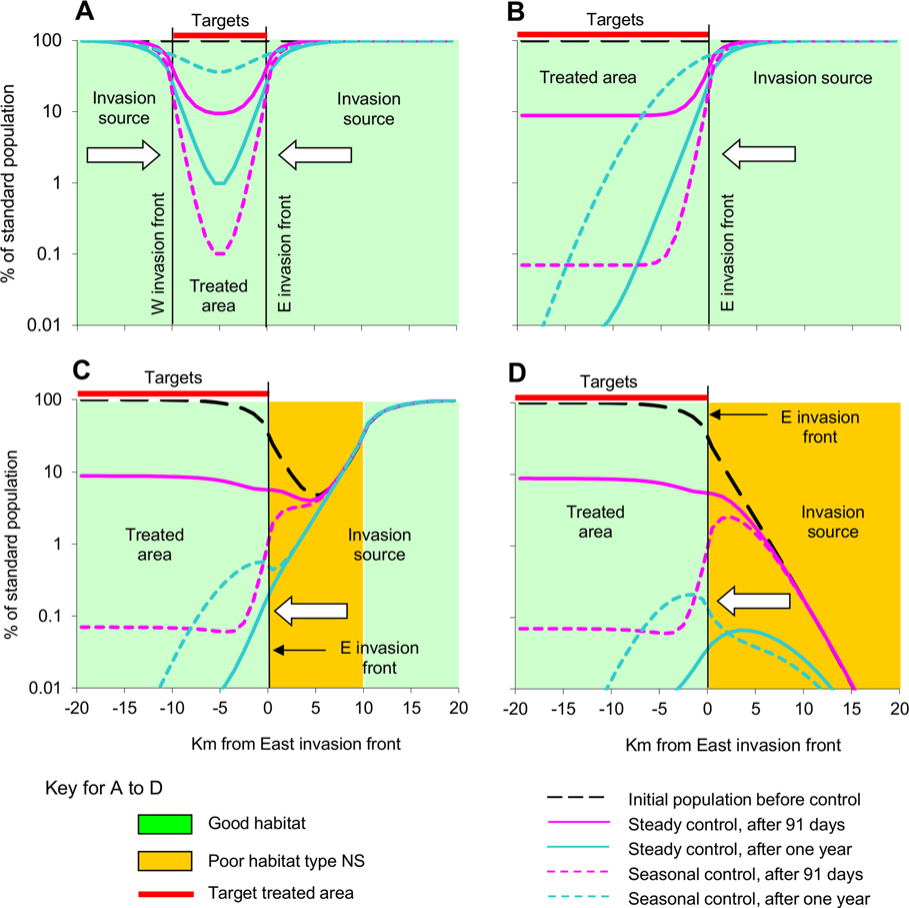
Population distribution at various times after starting steady or seasonal control in good non-isolated habitat, with various habitats in the invasion source. Population density of tsetse, as a percent of the standard density, at various distances from the invasion front, at different times after the start of normal steady or standard seasonal control, in an area where the invasion source was all good habit adjacent to a treated area spanning 10km (A) and >40km (B), or where the treated area spanned >40km of good habitat and the invasion source had a 10km (C) and >40km (D) segment of poor habitat type NS. For normal steady control the daily kill rate was fixed at 5%. For standard seasonal control the rate was 15% for the first three months, followed by a linear decline to zero in the next three months. White arrows indicate the direction of invasion.

**Fig 6:**
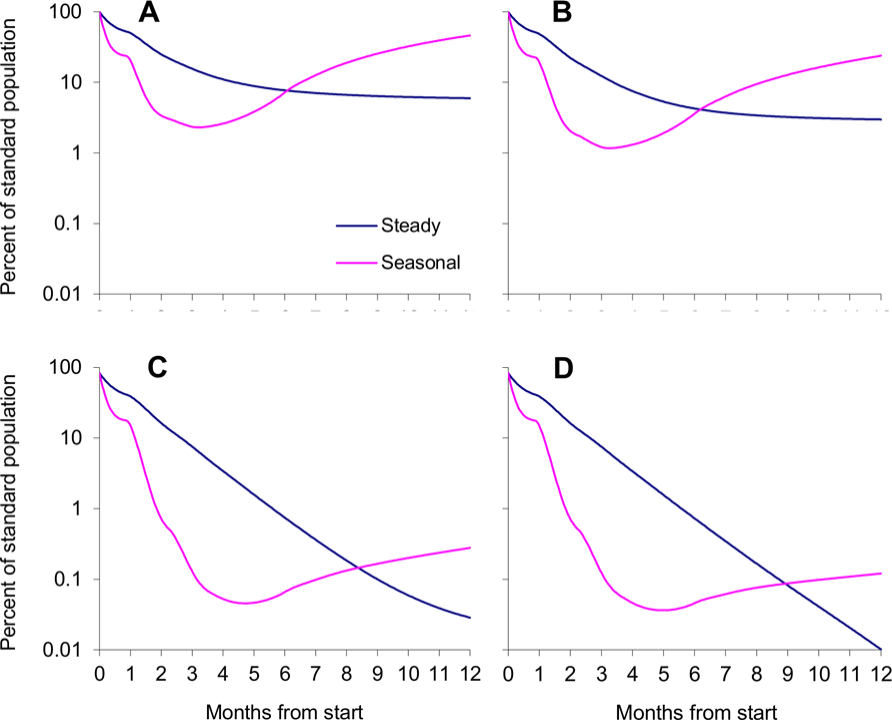
Population density at various times after starting steady or seasonal control in good non-isolated habitat, with various habitats in the invasion source. Average population density of tsetse in the treated area within 10km of the invasion front(s), as a percent of the standard density, at various times after the start of normal steady control or standard seasonal control, in an area where the invasion source was all good habit adjacent to a treated area spanning 10km (A) and >40km (B), or where the treated area spanned >40km of good habitat and the invasion source had a 10km (C) and >40km (D) segment of poor habitat type NS. For normal steady control the daily kill rate was fixed at 5%. For standard seasonal control the rate was 15% for the first three months, followed by a linear decline to zero in the next three months. The horizontal dotted line shows the 90% level control.

The sustained pressure was considered to come from self-sustaining populations in good habitat or poor habitat of type S, in a variety of arrangements. These led to distinctive abundances and distributions of the untreated tsetse population, and complex patterns in the degree of control in space (Fig. 5) and time (Fig. 6).

#### Good habitat as invasion source

It was taken first that the intervention area was a target-treated strip just 10km long, consisting of good habitat connected to large expanses of other good habitat as invasion sources at each end. This ensured that before control the abundance of tsetse was the same throughout the intervention area and the invasion sources (Fig. 5, A). No targets were placed in the invasion sources. With standard steady control the average density of tsetse within the 10km intervention area declined rapidly at first (Fig. 6, A), almost as fast as with an isolated population (Fig. 3). However, the population density soon started to level off towards a stable level dictated by the balance between the influx of tsetse from the invasion sources and the deaths imposed by targets. The movement of flies out of the invasion sources, coupled with the relative scarcity of flies available to move into these sources from the intervention area, meant that the abundance of tsetse was reduced a little in the proximal parts of the invasion sources. With standard seasonal control the high initial rate of kill produced, not surprisingly, a greatly enhanced rate of initial decline within the intervention area. However, the population recovered rapidly once the efficacy of the targets began to decrease after three months, and after a year the tsetse distribution was almost the same as in the starting situation (Fig. 5 A and Fig. 6 A). The common problem with both control policies was that the closeness of the invasion sources and the abundance of tsetse within them meant that the invasion pressure was particularly strong, so that the degree of control was above or hardly below the critical level in much of the intervention area for much of the time.

The degree to which the above effects were evident varied when there was a change to the length of the intervention area, and/or an alteration to the mobility of the flies. For example, the data of Fig. 5 A can be made to apply to double or half the standard mobility of the flies if the scale is adjusted so that the length of intervention area is double or half that shown, *i*.*e*., 20km or 5km, respectively. When the length of intervention area was kept at 10km the degree of population reduction due to either steady or seasonal control declined as the mobility of the flies increased, but there was relatively little effect of mobility on the relative performances of the two control policies (Table 3). Not surprisingly, seasonal control was always the better policy for giving a rapid three-month reduction in populations, and steady control was always the better when reduction was measured at the end of a year. When the population density was measured as an average throughout the year, seasonal control gave its best relative performance when fly mobility was lowest.

**Table 3:** Effect of mobility of tsetse on the population remaining after various periods of seasonal and steady control with sustained invasion pressure.

Measurement period	Control type	Mean daily displacement, m/day		
		150	300	450
**After three months**	**Steady**	12.3%	15.6%	18.8%
	**Seasonal**	1.2%	2.3%	3.5%
**After one year**	**Steady**	3.2%	5.9%	8.8%
	**Seasonal**	27.5%	46.0%	59.0%
**Average during year**	**Steady**	13.0%	15.8%	18.6%
	**Seasonal**	11.5%	17.6%	23.0%

Population density, as a percent of standard density, measured at various times after the start of normal steady control or standard seasonal control in a 10km strip of good habitat adjoining invasion sources of good habitat. All figures refer to the average density within the whole 10km strip.

Subsequent modelling returned to the standard mobility, but the above data should be taken as indications of how much a change in the mobility of the flies can affect all of the results.

#### Increased length of intervention area

For this scenario it was taken again that the habitat was uniformly good, but the length of the intervention area was increased greatly, to say ≥50km, so that the invasion sources were remote from most of it. The tsetse population in the middle of the intervention area was effectively isolated from the populations at the invasion fronts, at least for the few years needed for populations to diffuse over the great distances involved. Hence, in the centre of a large intervention area a year of applying the normal steady control or the standard seasonal resulted in >99.99% reduction of tsetse. The more interesting concern at present is what happened due to invasion at the edges of the large intervention area.

Allowing that the large intervention area and its invasion sources consisted entirely of good habitat, the two invasion fronts could be taken as similar so that it was necessary to consider only one (Fig. 5 B). The results obtained by the standard steady control and the standard seasonal control (Fig. 5 B and Fig. 6 B) were far better than when the target-treated area was just 10km long (Fig. 5 A and Fig. 5 B) since the invasion was occurring effectively from only one direction, not two, but the performance of steady control relative to seasonal control was much the same.

#### Poor habitat S as invasion source

The situation was not changed much if instead of having good habitat in all of the invasion source, as above, the habitat there was entirely of poor type S (Fig. 1 A). In that case, the percent population remaining in the first 10km of the intervention area after 91 days of control was 9.1% with standard steady control and 0.6% with standard seasonal control. At one year after control started the figures had virtually reversed to 0.6% and 6.0%, respectively. The self-sustaining populations in the invasion source and the intervention areas were reduced substantially by both control methods but were not eliminated.

### Partial buffers to sustained invasion pressure

In the above simulations the invasion sources with self-sustaining populations of tsetse were immediately adjacent to the intervention area, so that the intervention area received the full invasion pressure. We explored what happened when the self-sustaining population in the invasion source was separated from the intervention area by poor habitat forming a partial buffer to invasion. Such situations can be produced by fragmented habitats along the length of the river [20,24]

#### Poor habitat NS as buffer

The principles involved are illustrated by Fig. 5 C in which the good habitat of the intervention area is next to a 10km-long section of poor habitat NS, as a partial buffer to strong invasion pressure from good habitat to the East. Prior to treatment, the immigrants needed to sustain the tsetse population in the buffer zone came half from the good habitat in the East and half from the intervention area. With such a buffer the standard and seasonal control measures gave a more rapid decline in the tsetse population near the invasion front, the extent of invasion was reduced, and the recovery of the population after the cessation of the seasonal control was slower (Fig. 5 C and Fig. 6 C). The benefits of the buffer zone varied substantially according to its length. For example, with buffer zones of 5km, 10km and 15km long the percent population in the first 10km of the target-treated area after a year of standard steady control was 0.25%, 0.03% and 0.01%, respectively. At the end of the year in which standard seasonal control began the figures were 2.21%, 0.28% and 0.13%, respectively.

#### Very poor habitat as buffer

When the poor habitat of the 10km buffer zone was worsened to be very poor, so that all death rates of tsetse in it were treble, not double, the standard levels, the invasion pressure was reduced and the degree of control was improved. For example, the average population density in the first 10km of the intervention area at the end of a year of standard steady control was 0.029% when the habitat of the buffer zone was poor, as against 0.005% when the habitat there was very poor. With standard seasonal control the figures at the end of the year were 0.278% and 0.009%, respectively. Hence, with very poor habitat in the buffer zone there was little difference between the performances of steady and seasonal control.

### Non-sustained invasion pressure

In all of the above simulations tsetse always remained in the invasion source and could invade once control stopped, so being able to restore eventually the pre-treatment levels of infestation. The only hope of a permanent end to the invasion problem occurred when the arrangement of poor habitat ensured that the sustainability of the population in any part of the invasion source depended on immigrants that came entirely from the intervention area. With such an arrangement, a year of standard steady control in the good habitat was just sufficient to bring the tsetse population there to its collapse point (Fig. 6, D). Tsetse still remained in the invasion source then (Fig. 6, D), but at a density too low to give an invasion pressure that was sufficiently strong and sustained to re-establish a population in the good habitat. Hence, if all control halted after a year of standard steady control the population density in the 10km on either side of the invasion front dropped naturally to <0.01% after another 16 months, at which time the tsetse population could not recover even if the poor habitat were improved by, say, an increase in host numbers or an improvement in vegetation.

If the poor habitat in the invasion source were worsened by allowing that all death rates there were three times the standard levels, the initial population density in the first 5km of the invasion source was reduced to an average of 8% (range 1–23%) of the density in the first 5km of the good habitat. In this scenario the population in the first 10km of the intervention area dropped to <0.01% on day 329 of standard steady control and day 123 of standard seasonal control. In the first 10km of the invasion source the population declined to this level on days 247 and 164, respectively. Allowing that overall elimination could not be regarded as achieved until population densities in both the invasion source and the intervention area were reduced beyond possible recovery, the steady control was effective in 11 months and the seasonal control in half that time.

### Targets barriers to invasion

Targets used as barriers to invasion are most economical and effective when deployed in the invasion source at densities recommended for tsetse elimination [12]. Hence, in the present work the target deployments intended to simulate barriers were extensions of the deployments in the intervention area.

#### Barriers against sustained pressure

When the tsetse population in the invasion source was self-sustaining, creating permanent invasion pressure, the target barrier in the invasion source had to be operated each year. Moreover, when the invasion source and the intervention area consisted entirely of good habitat the barrier targets had to be deployed far into the invasion source, especially if the targets were effective only seasonally. For example, with standard seasonal control the deployments had to extend 16km into the invasion source, as against 11km with normal steady control. If the proximal part of the invasion source were poor habitat NS, occurring as sections 5, 10 and 15km long interposed between good habitats in the intervention area and in the more distal invasion source to the East, the required extensions of deployments were 15, 13 and 10km, respectively, for standard seasonal control. The figures for normal steady control were lower, at 10, 7 and 1km, respectively.

#### Barriers against non-sustained pressure

For those scenarios involving a non-sustained pressure of invasion, from sources with populations that were not self-sustaining, it was not absolutely necessary to put targets in the invasion source since the population there would expire once the flies disappeared from the intervention area. Nevertheless, the speed of eliminating the whole infestation, covering the intervention area and the invasion source, could be speeded greatly by extending target deployments to embrace the first few kilometres of the invasion source. For example, with the invasion source consisting entirely of poor habitat NS (Fig. 5, D) the extension of target deployments into the first 3km ensured that the standard steady control reduced the population everywhere to <0.01% in 341 days, thus avoiding the previous need for much work in the second year.

There was no benefit in extending the standard steady target treatment for more than 3km into the invasion source since with the greater extensions the limiting factor was the speed of controlling the population in the target-treated area well inside the good habitat, not the speed of reducing the population in the invasion source. However, with the standard seasonal treatment it was much more important to reduce the potential invaders quickly. Hence, there was substantial benefit in extending the standard seasonal deployments to well inside the invasion source. For example, when the extension reached to 3, 6 and 9km into the source the population everywhere dropped to <0.01% in 308, 167 and 119 days, respectively.

## Discussion

We used a deterministic model to explore the ways that the abundance and composition of tsetse populations change according to whether the population can or cannot sustain itself in the absence of immigration from other populations nearby. We then simulated the effect of controlling tsetse in operational areas consisting of one or more habitats with distinctive types of population, using targets deployed in all or part of the infestation and operated either continuously at relatively low density, or at higher density for a few months.

### Validity

The present model was basically the same as the Tsetse Muse model that appears to perform well in predicting the impact of control measures against self-sustaining populations of tsetse existing in isolated and non-isolated situations [21]. It is hardly surprising that good predictions of such matters are possible since models can be fed with much reliable detail about the dynamics of self-sustaining populations [22]. The salient features of such dynamics are that the natural daily death rates of adult tsetse cannot exceed about 3% per day and that the rates need to be increased by only about 1% in order to cause a population to expire [28]. For most of the time the actual death rates imposed by control measures are several times greater than this, so swamping any error in the model's understanding of the natural death rates and their degree of density dependence. Hence, present indications for the effects of steady or seasonal use of targets against naturally self-sustaining populations appear valid.

The validity of models is more questionable when simulating the natural sustainability of tsetse in various habitats in the invasion sources since this can depend on relatively slight changes in natural death rates and the patterns and extents to which such changes are modified by density dependence. Nevertheless, the present modelling suggests the various types of natural phenomena and the control results that can occur when populations near the operational area are or are not self-sustaining, without arguing about exactly what levels of natural mortality actually occur in various habitats and at different population densities.

### Seasonal versus steady control

In many situations it is likely to be cheaper and more convenient to operate tiny targets seasonally, at high density for a short time, as against the normal use of relatively few targets for extended periods. Hence, seasonal control would be the preferred option provided its efficacy were at least as good as steady control. In general, since the seasonal use of targets imposes the same pattern of mortality as the seasonal spraying of habitat with residual insecticides, the huge successes achieved by such spraying [29,30] enhance greatly the credibility of seasonal targets. Nevertheless, present work shows that the relative efficacy of seasonal as against steady use of targets depends on the aims and location of operations.

In operational areas that are free of any invasion, and where the most sensible aim is local elimination, the seasonal control can be advantageous in eliminating the flies in about a third or half of the time required by the normal steady use of targets. This could be particularly advantageous in countering a rapidly growing epidemic of sleeping sickness. Where the operational area is not entirely isolated, seasonal control could be highly beneficial if there were the sort of seasonal isolation in which the entire tsetse population concentrates in the operational area at certain seasons, as was commonly exploited in seasonal spraying campaigns [29,30]. In other cases where the invasion problem is not seasonal it can be feasible to aim for elimination provided the tsetse population in the invasion source is not self-sustaining, but in this case the seasonal work can be disadvantageous in allowing relatively little time for the population in the invasion source to expire.

In yet other situations where the invasion pressure is maintained all year round by a self-sustaining population in the invasion source, so that only feasible aim is the partial reduction in tsetse abundance, not elimination, there may be little to choose between the efficacies of seasonal and steady control in respect of the mean reduction of tsetse densities during the year. It is mainly the annual pattern of reduction that differs. Seasonal control gives the greatest initial reduction, but then allows the flies to recover so that at the end of a year the population density is least with steady control. It is only in respect of target barriers that seasonal control seems markedly less effective than steady control.

### Degree of self-sustainability

Present simulations show that in choosing the location and aims of operations, and then deciding whether to adopt steady or seasonal deployments, it is important to assess quickly the degree to which various habitats in or near the operational area contain self-sustaining populations of tsetse. Such an ability is especially important since it bears also on the relative feasibility of other control measures that differ in their time frames and potential location, such as steady control by insecticide-treated cattle [31] and seasonal control by various sorts of insecticidal sprays [32]. For example, the fuller consideration of this matter might have avoided the aerial spraying of several thousand square kilometres on the margins of the main infestations in Zimbabwe in the 1980s [32]. In other infestations of the region the populations on the edge were not self-sustaining and needed no direct treatment [19,23].

In some cases a decision on sustainability is easy. For example, any population, even a very sparse one, can be taken as self-sustaining if it has inhabited an isolated habitat for several years with no signs of population decrease or habitat degradation. The more difficult problem occurs when poor habitat with persistent tsetse adjoins other habitat where population densities are much greater, so raising uncertainty as to whether the population of the poor habitat is critically dependent on immigration. Correlations of the presence of tsetse with various habitat features can be of background interest [20,33], but it is more important to understand the dynamics of the tsetse population in various parts of the overall infestation, so explaining why tsetse are present in each place. Unfortunately, the production of suitable data for the births and deaths of tsetse in just one isolated type of habitat is daunting enough [22], so that full investigation of tsetse dynamics, including movement, in several contiguous habitats is beyond reasonable expectation.

Against this, present simulations suggest that relatively simple inspection of tsetse catches, to assess the abundance and composition of the tsetse population in different places might offer useful clues. It seems that non-self-sustaining populations could be identified not only by being sparse, but also by showing: (i) densities that decline continuously on moving further from the better habitat, (ii) high mean ages of females and (iii) high proportions of females. It is difficult to find field data to confirm or refute these criteria since there are scant field data for sampling of populations that are known to be non-self-sustaining. Even such data that do exist refer to savannah tsetse that are not confined entirely to riverine habitat. Some of the most useful data are those produced by trapping *G*. *pallidipes* at Nguruman, Kenya, where for most of the year the flies are partially restricted to good habitat composed of riverine forest [34] seemingly because the more open habitat further from the river limits the outward diffusion of tsetse by providing reflective contacts. About 10km of the river was treated with traps for a year, at a density sufficient to eliminate the flies if there had been no invasion [11]. However, invasion meant that the flies were not in fact eliminated but showed apparent densities that had roughly stabilized after a year, at around 1–5% of the pre-treatment level. In keeping with current expectations for a non-self-sustaining population that has approached stability, the percent of females in catches was then very high, at about 80%, with increased proportions of old females [11].

Perhaps the greatest problem in using catches to assess the sustainability of tsetse populations is that in many places where the population is suspected of being unable to sustain itself it is difficult to catch enough tsetse for satisfactory studies. However, very low catches suggest a sparse population under severe stress and so offer *prima facie* evidence for a lack of self sustainability. Thus, while scientific research in such situations should be encouraged as much as possible, most of the demand at present might be for bold action to build up an empirical body of knowledge of what works satisfactorily in various situations. This empirical approach might not be elegant scientifically, but its usefulness has been demonstrated many times in the past, as in helping the evolution of highly effective policies for control by insecticidal sprays [30].

In conclusion, the seasonal use of tiny targets deserves field trials. Whether control is seasonal or steady, involves targets or any other method, and irrespective of what tsetse species is tackled, there could be much benefit in improving our ability to identify populations that are not self-sustaining.
